# Notes and News

**Published:** 1905-03-04

**Authors:** 


### NOTES AND NEWS.

It is proposed to erect a cottage hospital at Malton, in Yorkshire. The Hon. G. Dawnay has been elected chairman of the Monthly Board.

A new Orthopædic Hospital has been opened in Upper Merrion Street, Dublin. There are four large and four small wards, and accommodation for 56 patients is now ready. The hospital can contain 100 patients, but all the wards are not available at present.

A new wing has been opened at the Aberdeen Maternity Hospital. It consists of a basement and ground floor. On the ground floor is a ward for 12 patients. There are besides bath-rooms, offices, and accommodation for the nursing staff. The estimated cost is about £2,000.

A meeting of the Ladies Association in aid of the North-Eastern Hospital for Children, Hackney Road, was held on Thursday at 19 Stratford Place to consider the arrangements for the luncheon at the Mansion House, at which the Lord Mayor has consented to preside, on May 22nd, for the purpose of raising £15,000 to enable the work of the hospital to be carried on free from debt during the present year, and to pay off the mortgage on the new building.

Mr. W. Johnson Smith, F.R.C.S., principal medical officer of the Seamen's Hospital Society, Greenwich, is about to retire after being a member of the surgical staff of that hospital for 35 years. In recognition of his long and eminent public service, the medical staff of the Seamen's Hospital Society propose to entertain him at dinner, and present him with a testimonial, on Wednesday, April 5th, at the Trocadero Restaurant. Sir Patrick Manson will take the chair at this interesting function, and those of the numerous friends of Mr. Johnson Smith who desire to be present and to join his colleagues in doing honour to the distinguished surgeon, should communicate with Kenneth Steele, Esq., care of Seamen's Hospital Society, 13a Cockspur Street, S.W., Honorary Secretary of the Dinner Committee.

It having been decided to establish a Consumption Sanatorium for Cork, two local medical men have been requested to advise of the site which will be advertised for.

The endeavour to prevent the large infantile mortality in the metropolis is spreading. Dr. Sykes, the medical officer of health for St. Pancras, has prepared a report on the subject, and the Borough Council have requested him to elaborate a scheme.

Alnwick is to have a new infirmary, and the result of a competition in which 20 architects competed, the plans of Messrs. Wrightman Douglas, of Alnwick and Newcastle, and of Messrs. Boyd and Groves, of Newcastle, were awarded the prize of £100 conjointly. The total cost of the scheme is not expected to exceed £6,000.

The question of tinkering the old buildings or providing a new workhouse infirmary at Plymouth is still under consideration. The Guardians should remember that sooner or later public opinion will demand a properly-arranged institution for the treatment of the sick poor and that the names of those who spent money on unsatisfactory patching up will be held up to censure when it is found necessary to undo the work they now contemplate undertaking at a considerable cost.

The seventy-third annual meeting of the British Medical Association will be held at Leicester from Monday, July 24th to Friday, July 28th, inclusive. This year it is proposed to extend the scope of the exhibition by reserving a section for motor-cars, carriages, etc., specially suitable for medical men. The Drill Hall, Newarke, Leicester, and the adjoining Magazine Square have been secured for the exhibition—a most convenient position, practically adjoining the Technical Schools where the scientific meetings of the Association will be held.

It is interesting to learn that the gentleman who has recently bequeathed £17,000 to the Leeds Infirmary, revealed no intention of his intention to benefit the institution during his lifetime. He lived very simply, was not known to be a rich man, nor did he exhibit any great generosity towards charitable institutions. He always, however, expressed his admiration for the Leeds Infirmary and its work. The stipulation which accompanied the gift is curious. The ward bearing his name is to contain a marble statue of the donor, and a copy of his will is to be displayed in a conspicuous position in the infirmary once a year.

We congratulate Mr. Thomas Hayes upon his election to the office of clerk to St. Bartholomew's Hospital. Mr. Hayes has held the secretaryship of the East London Hospital for Children for many years with credit to himself and great benefit to the institution. There were a large number of candidates for the clerkship. The election was keenly contested, and Mr. Hayes's success is popular and well deserved. Mr. Sydney Phillips, of St. Thomas's Hospital, writes to deny a "widespread rumour," to the effect that he was a candidate for the clerkship of St. Bartholomew's Hospital.

